# HLA diversity in ethnic populations can affect detection of donor-specific antibodies by single antigen beads

**DOI:** 10.3389/fimmu.2023.1287028

**Published:** 2023-11-23

**Authors:** Justin C. Quon, Kelli Kaneta, Nicholas Fotiadis, Jondavid Menteer, Rachel M. Lestz, Molly Weisert, Lee Ann Baxter-Lowe

**Affiliations:** ^1^ Department of Pathology, Keck School of Medicine, University of Southern California, Los Angeles, CA, United States; ^2^ Division of Nephrology, Children’s Hospital Los Angeles, Los Angeles, CA, United States; ^3^ Department of Pathology and Laboratory Medicine, Children's Hospital Los Angeles, Los Angeles, CA, United States; ^4^ Department of Pediatrics, Keck School of Medicine, University of Southern California, Los Angeles, CA, United States; ^5^ Division of Cardiology, Children’s Hospital Los Angeles, Los Angeles, CA, United States

**Keywords:** donor specific antibodies, HLA antibodies, HLA diversity, race and ethnicity, single antigen bead assays (SAB), solid organ transplantation

## Abstract

**Introduction:**

In solid-organ transplantation, human leukocyte antigen (HLA) donor-specific antibodies (DSA) are strongly associated with graft rejection, graft loss, and patient death. The predominant tests used for detecting HLA DSA before and after solid-organ transplantation are HLA single antigen bead (SAB) assays. However, SAB assays may not detect antibodies directed against HLA epitopes that are not represented in the SAB. The prevalence and potential impact of unrepresented HLA epitopes are expected to vary by ethnicity, but have not been thoroughly investigated. To address this knowledge gap, HLA allele frequencies from seven ethnic populations were compared with HLA proteins present in SAB products from two manufacturers to determine unrepresented HLA proteins.

**Materials:**

Allele frequencies were obtained from the Common, Intermediate, and Well Documented HLA catalog v3.0, and frequencies of unrepresented HLA types were calculated. Next-generation sequencing was used to determine HLA types of 60 deceased solid-organ donors, and results were used to determine if their HLA-A, -B, -C, and -DRB1 proteins were not present in SAB reagents from two vendors. Unrepresented HLA proteins were compared with the most similar protein in SAB assays from either vendor and then visualized using modeling software to assess potential HLA epitopes.

**Results:**

For the seven ethnic populations, 0.5% to 11.8% of each population had HLA proteins not included in SAB assays from one vendor. Non-European populations had greater numbers of unrepresented alleles. Among the deceased donors, 26.7% (16/60) had at least one unrepresented HLA-A, -B, -C, or -DRB1 protein. Structural modeling demonstrated that a subset of these had potential HLA epitopes that are solvent accessible amino acid mismatches and are likely to be accessible to B cell receptors.

**Discussion:**

In conclusion, SAB assays cannot completely rule out the presence of HLA DSA. HLA epitopes not represented in those assays vary by ethnicity and should not be overlooked, especially in non-European populations. Allele-level HLA typing can help determine the potential for HLA antibodies that could evade detection.

## 1 Introduction

Single antigen bead (SAB) assays play a key role in solid organ transplantation because they can be used to detect donor-specific antibodies (DSA) against human leukocyte antigens (HLA) which are associated with poor transplant outcomes (1, 2). Before transplantation, SAB assays are routinely used for virtual crossmatching and excluding incompatible donors. After transplantation, SAB assays are used to determine immunosuppressive regimens and monitor DSA which are associated with antibody-mediated rejection (AMR) and decreased graft survival. DSA can also be a challenge to hematopoietic stem cell transplantation if donors are HLA mismatched (3–5). Further, presence of DSA at transplant can cause rejection or impair engraftment (3–5).

The development of SAB assays for detecting HLA DSA was a major advancement (6), but SAB assays represent only a fraction of the vast HLA diversity of the human population. With more than 35,000 currently known HLA alleles, it is neither feasible nor cost-effective to test for antibodies against all possible HLA epitopes (7, 8). When a donor antigen has an epitope that is not present in any of the HLA proteins in the SAB assays, clinically significant DSA may be undetectable. Failure to detect HLA antibodies may have clinical consequences. Virtual crossmatching may be erroneous, or rejection can be improperly categorized in that antibody-mediated rejection may be overlooked. Since HLA allele frequencies and associations are different for each ethnic population, the ability of SAB assays to detect HLA antibodies is expected to vary by ethnic ancestry (9).

The prevalence of HLA proteins not found in SAB assays, the quantitative impact of ethnicity on HLA coverage by SAB assays, and the potential impact of unrepresented HLA epitopes have not been rigorously investigated. In this study, a multifaceted approach was used to examine the prevalence and potential immunogenicity of unrepresented HLA proteins and to assess how treatment guidelines could address the possibility of unique HLA epitopes.

On an international level, the antigen coverage of commercially available SAB assays from two vendors was determined for seven international ethnic populations as defined by Hurley et al. (10): Hispanic (HIS), Native American (NAM), African American (AFA), Asian and Pacific Islander (API), Middle Eastern and North Coast of Africa (MENA), European (EUR), and mixed or unknown ethnicity (UNK). On a local level, antigen coverage was determined for 60 deceased organ donors. In these donors, HLA proteins with epitopes unlikely to be detected by SAB assays were identified, and their potential immunogenicity was demonstrated using structural modeling. Additionally, a patient receiving a graft from a zero-ABDR mismatched donor allocated by the United Network of Organ Sharing (UNOS) was used to illustrate limitations in the use of low-resolution typing for determining histocompatibility and to demonstrate why allele-level HLA typing is important for determining the potential for DSA that are undetectable using SAB assays.

## 2 Materials and methods

### 2.1 Single antigen bead assays for HLA antibody detection and HLA allele frequencies

SAB assays from two manufacturers were studied. HLA types were obtained using product inserts for two SAB assay vendors: LABScreen™ Single Antigen Class I lots 4 and 12, Class II lots 4 and 13 (One Lambda Inc, West Hills CA), and LIFECODES^®^ Single Antigen Assays, Class I lot 3009675 and Class II lot 3009431 (Immucor Inc, Norcross GA). The frequencies of each of the corresponding HLA alleles in seven ethnicities were obtained from the Common, Intermediate, and Well Documented (CIWD) catalog v3.0.0 (10). Allele frequencies were categorized as common (≥ 1 in 10,000), intermediate (≥ 1 in 100,000), well documented (≥ 5 occurrences), or not CIWD as described by Hurley et al. (10). The term “frequency” here is used to refer to the allele frequency data published in the CIWD report, with the understanding that the CIWD catalog inherently suffers from variability in the resolution and accuracy of HLA typing data. Ethnicity was categorized based on CIWD classifications and used as a marker for genetic ancestry.

For every HLA protein in the SAB assays, the CIWD catalog was used to determine the corresponding allele’s frequency in each of the seven ethnic populations. Antigen frequencies were calculated from allele frequencies assuming Hardy-Weinberg equilibrium. The percentage of each ethnic population expressing an HLA protein not represented in a vendor’s SAB assays is then given by


$$\text{Percent Not Represented}=100\times \left(1-\sum_{i=1}^{k}f_{i}\right)$$

where $f_{i}$ is the frequency of antigen i in that ethnic population, and the range i=[1, 2, …k] is the set of all k antigens represented in the vendor’s SAB assay for a specific HLA locus. The percent representation of each vendor’s SAB assay was then stratified based on ethnicity and HLA locus.

### 2.2 Deceased donors and structural models

Deceased donors who had donated organs to any solid-organ transplant recipient at Children’s Hospital Los Angeles between 2018 and 2021 were included in this study if allele-level HLA-A, -B, -C, and -DRB1 genotypes had been determined using next generation sequencing (NGS) methods. Donor ethnicity was defined as the reported ethnicity to UNOS. HLA genotypes were determined using reagents and software purchased from Omixon (Budapest, Hungary) or CareDx (Brisbane, CA). Each donor allele was categorized based on whether the protein encoded by the allele was included in standard panels of SAB assays available from two commercial vendors: Vendor 1 and Vendor 2. This study was deemed exempt by the Children’s Hospital Los Angeles Human Subjects Protection Program and Institutional Review Board (CHLA-23-00143).

For every donor allele that was not represented in either vendor’s SAB assays, the most similar HLA protein in either SAB assay was determined based on similarity of the amino acid sequences (IPD-IMGT/HLA Database, v3.50) (7). Structural models (UCSF Chimera, RCSB Protein Database) were used to visualize the location of all amino acid differences between an unrepresented HLA protein and its corresponding similar HLA protein in the SAB assays. When crystal structures of the corresponding HLA protein were not available on the RCSB Protein Database, the closest available HLA protein of the same locus was used. To assess the likelihood that these HLA epitopes could be immunogenic, all amino acid differences were categorized based on their polarity, charge, and surface exposure. Determination of an epitope’s accessibility from the protein surface was performed by computational methods described by Kramer et al. (11).

### 2.3 Case study for a 0-ABDR mismatched donor

Low-resolution HLA types of a donor-recipient pair were obtained from UNOS. Allele-level typing at the HLA-A, -B, -C, -DRB1, and -DQB1 loci was determined using NGS. HLA Matchmaker (ABC v4.0 and DRDQDP v3.1) was used to determine HLA epitopes based upon eplet matching (12).

## 3 Results

### 3.1 HLA representation in single antigen bead assays

All ethnic populations have some HLA alleles that are not represented in SAB assays. However, HLA allele frequencies vary among different ethnic populations, and this impacts the likelihood that each population’s HLA alleles are represented in SAB assays. We examined reagents from two vendors that are routinely used to detect HLA DSA.

**Figure 1:**
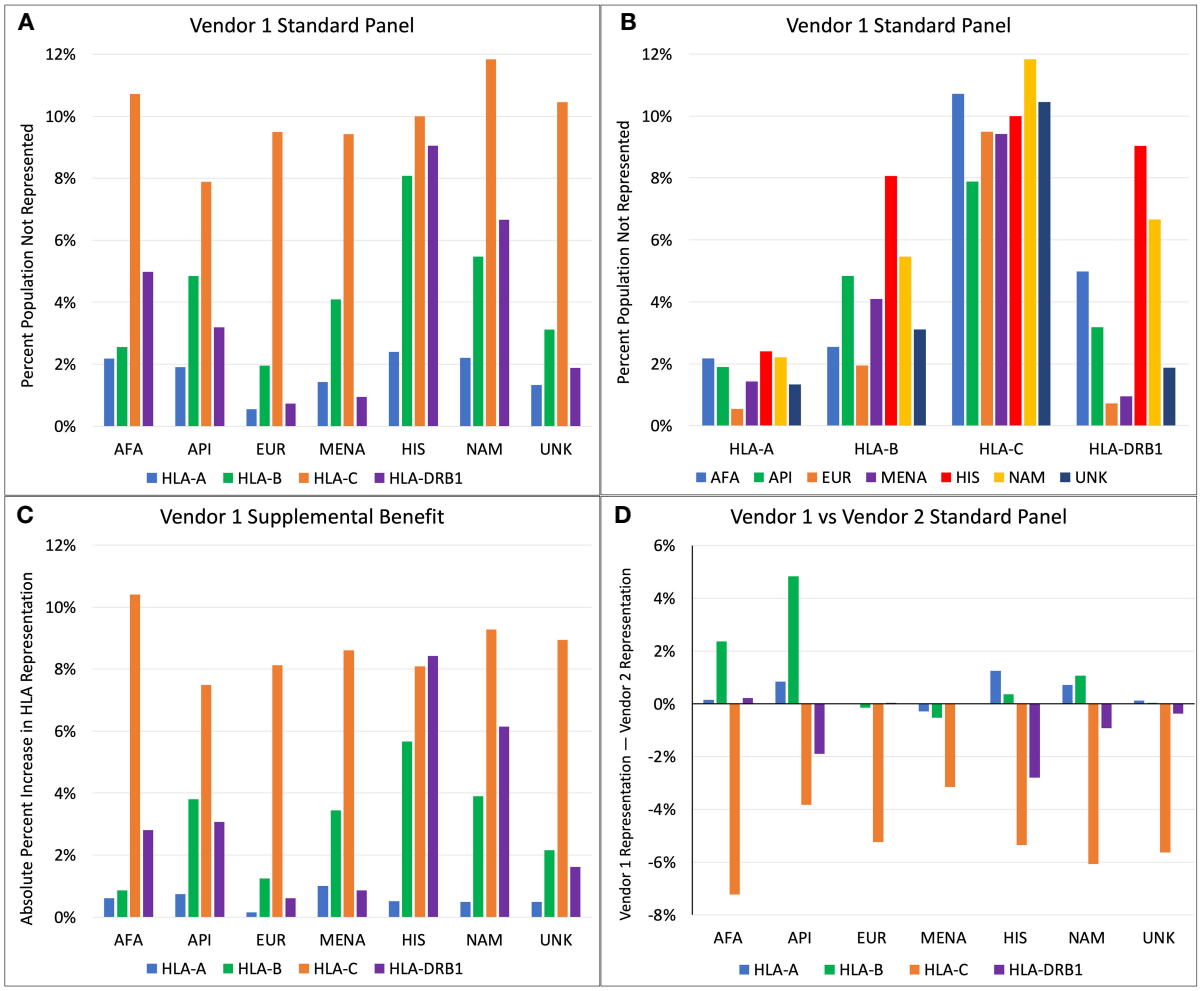
**(A)** Percent of population not represented by Vendor 1 standard panel, categorized by ethnicity. **(B)** Percent of population not represented by Vendor 1 standard panel, categorized by HLA locus. **(C)** Increase in absolute percent representation when combining Vendor 1 supplemental and standard panels compared to Vendor 1 standard panel only. **(D)** Comparison of percent representation between standard panels of Vendor 1 and Vendor 2 (Positive axis indicates better representation by Vendor 1).

For Vendor 1, the percentage of each population with HLA proteins from a given locus not represented by the standard SAB assays ranged from 0.5% to 11.8% (**Figures 1A, B**). HLA-A, -B, and -DRB1 antigen frequencies were best represented for the EUR population, where 0.5%, 2.0%, and 0.7% had antigens not represented in the SAB assay from Vendor 1 for each locus respectively (**Figure 1A**). The HIS and NAM populations were the most poorly represented at the HLA-A, -B, and -DRB1 loci. Roughly 2.4%, 8.1%, and 9.0% of the HIS population have HLA-A, -B, and -DRB1 alleles that are not represented in the standard SAB, respectively. When comparing representation by HLA locus, the standard panel of Vendor 1 represents the greatest proportion of HLA-A antigens, ranging from 0.5% to 2.4% of people not represented among all ethnic populations (**Figure 1B**). For HLA-C, the best representation was in the API population (7.9% unrepresented), and the lowest representation was seen in the NAM population (11.8% unrepresented). The HLA-C locus had the poorest representation among all populations, with 7.9% to 11.8% of the populations expressing unrepresented HLA antigens.

Addition of the supplemental panel from Vendor 1 to the standard panel yielded variable benefits in percent representation depending on ethnicity and HLA locus (**Figure 1C**). The increase in absolute percent representation with the supplemental panel ranged from 0.1% to 10.4%. Compared to other HLA loci, representation at HLA-C increased most drastically for all races except for the HIS population, with up to a 10.4% increase in HLA-C representation for the AFA population. At the HLA-A locus, the supplemental panel benefited the MENA population most (1.0% increase), while the EUR population had the lowest increase in percent representation (0.1% increase). This is an expected finding because only 0.5% of the EUR HLA-A alleles were unrepresented in the main Vendor 1 panel. The supplemental panel increased HLA-B representation the most for the HIS, API, and MENA populations, with a 5.7%, 3.8%, and 3.4% increase in percent representation, respectively. The greatest benefits at the HLA-DRB1 locus were seen in the HIS and NAM populations, with an 8.4% increase in percent representation for the HIS population and a 6.1% increase for the NAM population.

**Figure 1D** compares HLA representation between the standard SAB assays of Vendor 1 and Vendor 2. At the HLA-A locus, Vendor 1 provides marginally better representation for the HIS population with a 1.2% increase in percent representation compared to Vendor 2. All other differences at the HLA-A locus changed by less than 1% representation. Differences in HLA-B representation are greatest for API and AFA populations, where Vendor 1 has 4.8% better coverage for API and 2.4% better coverage for the AFA population. For all ethnicities, the standard panel of Vendor 2 better represents HLA-C antigens, with the increase in percent representation ranging from 3.1% for the MENA population to 7.2% for the AFA population. Vendor 2 better represents the HLA-DRB1 antigens for most populations. The greatest benefit for HLA-DRB1 coverage was seen in the HIS and API populations, with an additional 2.8% and 1.9% coverage compared to Vendor 1, respectively.

### 3.2 Deceased donors and structural models

The ethnic breakdown of the 60 deceased donors is as follows: 19 Caucasian, 24 Hispanic, 4 African American, 1 Asian, 2 mixed, and 8 unknown (**Table 1**). A total of 467 HLA-A, -B, -C, and -DRB1 alleles were observed among the 60 donors. There were 126 unique alleles at allele-level resolution (**Table 2**). Five alleles were not designated as “common” in any ethnic population. Of these five alleles, there were three intermediate alleles (HLA-B*35:43, B*35:116, and DRB*16:12), one well documented allele (HLA-A*02:164), and one allele that was not CIWD (HLA-B*40:225). None of these five alleles were represented in SAB assays from either Vendor 1 or Vendor 2.

**Table 1:** Ethnic breakdown of deceased donors.

Ethnicity	Donors (n = 60)	Recipients (n = 62)^a^
Caucasian	19 (31.7%)	9 (14.5%)
Hispanic	24 (40.0%)	37 (59.7%)
African American	4 (6.7%)	4 (6.5%)
Asian	1 (1.7%)	3 (4.8%)
Mixed	2 (3.3%)	0 (0%)
Unknown	9 (13.3%)	9 (14.5%)

a Organs from a single donor were transplanted into multiple recipients.

**Table 2:** Allele distribution in deceased donors.

	HLA-A	HLA-B	HLA-C	HLA-DRB1	Total
Count					
Total	113	119	117	118	467
Unique	27	48	19	32	126
CIWD Frequency^a^					
Common	112	116	117	117	462
Not Common	1	3	0	1	5
Intermediate	0	2	0	1	3
WD	1	0	0	0	1
Not CIWD	0	1	0	0	1
Vendor Representation					
Both standards	100	93	92	87	372
One standard	6	5	18	16	45
Supplement only	2	12	4	11	29
Not in either vendor	5	9	3	4	21

a Using highest CIWD frequency among all seven ethnic populations.

A total of 26.7% (16/60) donors had at least one HLA-A, -B, -C, or -DRB1 allele whose protein products were not represented in SAB assays from either vendor. Among the 21 (20 unique) alleles that were not represented in either vendor’s SAB assays, there were 16 common alleles, 3 intermediate alleles, 1 well documented allele, and 1 allele that was not CIWD. Five of these alleles were HLA-A, 9 were HLA-B, 3 were HLA-C, and 4 (3 unique) alleles were from the HLA-DRB1 locus (**Table 3**). **Table 2** shows that 9.6% (45/467) of alleles were found only in one vendor’s standard panel, and 6.2% (29/467) of alleles were found only in the supplemental panel from Vendor 1.

**Table 3:** Unrepresented HLA proteins in deceased donors and properties of their amino acid mutations.

Unrepresented Antigen	Most Similar Represented	Δ Amino Acids	Δ Charge	Amino Acid Exposed
A*02:11	A*02:01	T73I	No	Yes
		H74D	Yes	No
A*02:164	A*02:01	M138L	No	Yes
A*23:17	A*23:01	H283P	No	TM^a^
A*24:25	A*24:02	Y7C	No	No
A*68:05	A*68:01	Q70H	No	Yes
		D74H	Yes	No
B*27:02	B*27:05	D77N	Yes	Yes
		T80I	No	Yes
		L81A	No	No
B*35:17	B*35:01	R97S	Yes	No
		L103V	No	No
B*35:23	B*35:01	Y99F	No	No
		I94T	No	No
		I95L	No	No
		L103V	No	No
		V152E	Yes	Yes
		L156W	No	No
		V194I	No	Yes
B*35:116	B*35:01	G120D	Yes	Yes
B*40:08	B*40:02	E63N	Yes	No
		S67F	No	No
B*40:11	B*40:02	S97R	Yes	No
B*40:225	B*40:02	S97N	No	No
B*44:05	B*44:02	D116Y	Yes	Yes
C*07:06	C*07:01	M307K	No	TM^a^
		A324V	No	CT^a^
C*07:18	C*07:01	A324V	No	CT^a^
C*15:09	C*15:02	L116S	No	Yes
DRB1*11:02	DRB1*11:03	F67I	No	Yes
DRB1*11:06	DRB1*11:04	V85A	No	Yes
DRB1*16:12	DRB1*16:02	R13K	No	Yes

a TM, transmembrane domain; CT, cytoplasmic tail.

**Table 3** lists all unrepresented HLA proteins that were identified in the sample of 60 deceased donors, as well as the protein in either vendor’s standard panel with the most similar amino acid sequence. Structural models were created for all proteins that were not represented in the SAB assays, with the exception of those with amino acid differences in the transmembrane domain or cytoplasmic tail (**Supplementary Figures 1**–**3**).

**Figure 2:**
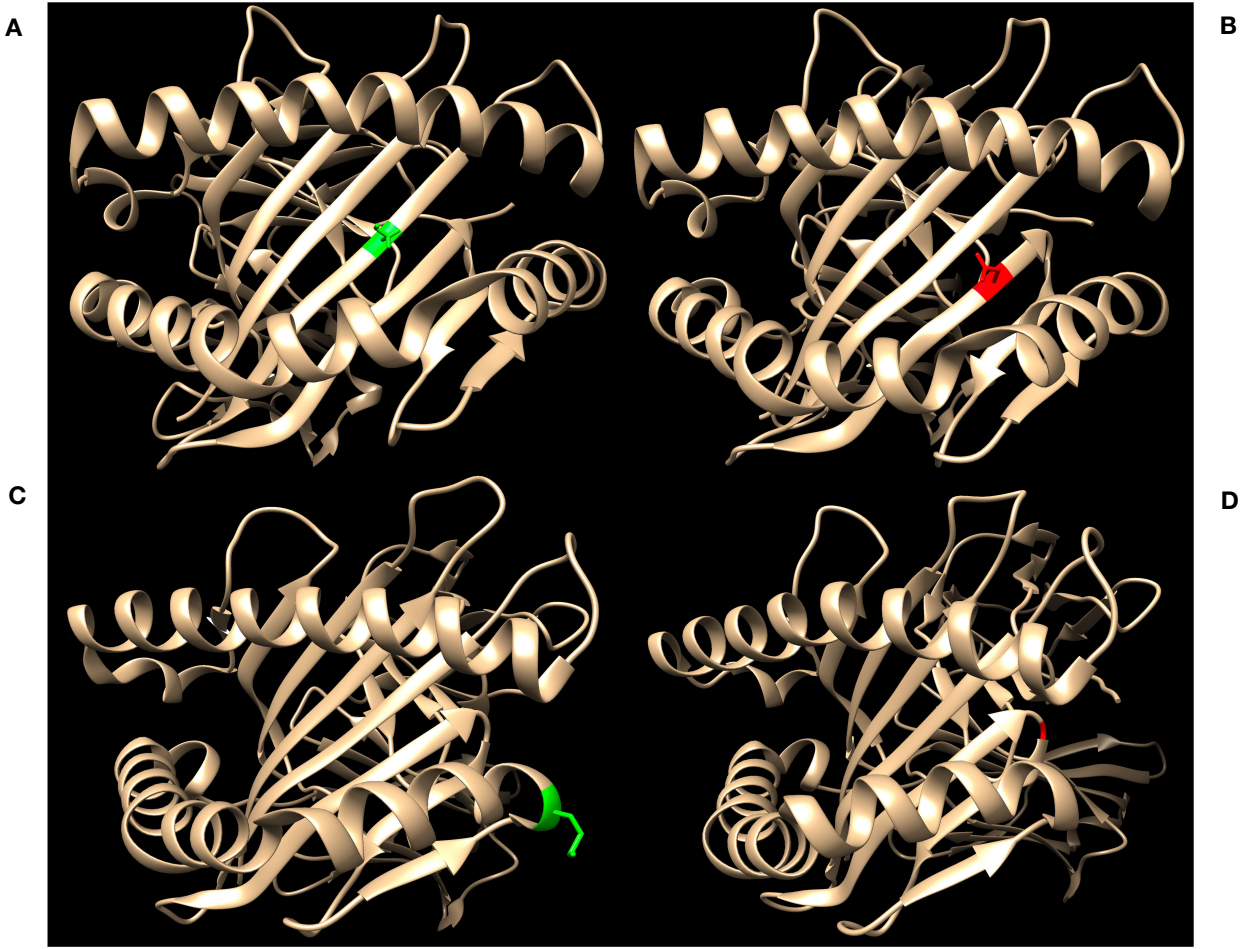
Green residues indicate no charge difference and red residues indicate a net charge difference compared to mutations of the corresponding unrepresented proteins. **(A)** Structure of HLA-B*40:02 with position 97S highlighted in green (RCSB 5IEK), corresponding to the unrepresented HLA-B*40:225. **(B)** Structure of HLA-B*44:02 with position 116D highlighted in red (RCSB 1M6O), corresponding to the unrepresented HLA-B*44:05. **(C)** Structure of HLA-A*02:01 with position 138M highlighted in green (RCSB 4U6Y), corresponding to the unrepresented HLA-A*02:164. **(D)** Structure of HLA-B*35:01 with position G120 highlighted in red (RCSB 2H6P), corresponding to the unrepresented HLA-B*35:116.

**Figure 2** highlights key examples of unrepresented proteins with varying immunogenicity and estimated likelihood of DSA detection by SAB assays. **Figure 2A** shows the structure of the reagent protein B*40:02, where the corresponding unrepresented B*40:225 protein has an S→N mutation at position 97. Because this mutation is both inaccessible to the antigen surface and unchanged in charge or polarity, it is less likely that DSA may develop against this amino acid difference.

In contrast, HLA-B*44:05 differs from its corresponding reagent protein B*44:02 by a D116Y mutation (**Figure 2B**). This mutation results in a net charge difference at physiological pH and the location is exposed to the antigen surface, which indicates a strong potential for generation of HLA antibodies against this epitope. However, 116Y is found in other HLA-B proteins in SAB assays, opening the possibility that antibodies against the 116Y site of HLA-B*44:05 may possibly be detected even if B*44:05 itself is not included in SAB assays.

Finally, the unrepresented A*02:164 differs from the reagent protein A*02:01 by a M138L mutation (**Figure 2C**). Likewise, B*35:116 differs from B*35:01 by a G120D mutation with a charge difference (**Figure 2D**). In these two cases, position 138 for the HLA-A locus and position 120 for the HLA-B locus are exposed to the cell surface and may be potential epitopes for development of HLA DSA. However, neither 138L (for HLA-A) nor 120D (for HLA-B) are found in any other proteins of the same locus in the SAB assays. Therefore, clinically significant HLA DSA may develop against these epitopes and if so, would likely not be detected by SAB assays.

### 3.3 Case study for a 0-ABDR mismatched donor

A patient who received a 0-antigen mismatch organ was studied to illustrate that it can be possible to have undetectable DSA when donors are apparently well-matched. A 5-year-old boy with end stage renal disease secondary to nephrotic syndrome received a kidney allograft from a deceased donor in March 2020. Using UNOS criteria, the donor was determined to be 0-ABDR mismatched with the recipient. However, retrospective NGS sequencing and subsequent analysis by HLA Matchmaker demonstrated multiple HLA mismatches at allele-level resolution (**Table 4**) and 7 eplet mismatches. Additionally, one of the donor’s mismatched HLA types (HLA-DRB1*35:12) is not included in standard panels from either vendor of SAB assays.

**Table 4:** HLA types of 0-ABDR mismatched donor-recipient pair by low-resolution typing and next-generation sequencing.

Type	HLA-A		HLA-B		HLA-C		HLA-DRB1		HLA-DQB1	
NGS	02:06	24:25	35:43	39:06	07:02	15:02	04:07	14:06	03:01	03:02
UNOS	2	24	35	39	7	15	4	14	7	8
NGS	02:01*	24:02*	35:43	35:12*	01:02*	04:01*	04:07	04:03*	–	03:02
UNOS	2	24	35	35	1	4	4	4	–	8

^*^HLA mismatches identified by retrospective NGS typing.

## 4 Discussion

The primary objectives of this study were to determine the percentage of HLA proteins in the population that are not represented in SAB assays, and how ethnicity impacts this percentage. All ethnic populations had unrepresented HLA proteins. SAB assays will not detect antibodies directed against HLA epitopes that are not represented in the SAB. This is especially important when donor populations are predominantly non-Europeans, where rates of unrepresented HLA proteins are higher. Ethnic differences in donor populations may potentially impact not only pre-transplant screening, but also post-transplant surveillance and rejection treatment. Failure to consider the possibility of unrepresented HLA proteins may potentially lead to incorrect assumptions about the absence of DSA.

The two vendors’ standard SAB panels have different strengths in covering the range of HLA types in ethnic populations, which may impact the ability to effectively screen potential recipients for HLA DSA before and after transplant. For vendor 1, 0.5% to 11.8% of the HLA-A, -B, -C, and DRB1 proteins were missing from the standard SAB assay, but this decreased to 0.1% to 2.6% when the supplemental panel was added. Vendor 1 representation for HLA-A, -B, and -DRB1 proteins was the best for the EUR population. When comparing the two vendors’ standard SAB panels, Vendor 2 had increased representation for HLA-C and HLA-DRB1 proteins for nearly all ethnic populations. Despite these relatively low missing fractions, greater numbers and frequencies of missing antigens are seen when considering a donor of a specific non-EUR ethnicity. In our transplant center, 26.7% of donors had unrepresented HLA-A, -B, -C, and -DRB1 proteins, and this percentage would increase substantially if heterodimeric proteins such as HLA-DQ and HLA-DP were considered.

**Figure 1** shows that 7.9% to 11.8% of the population have HLA-C antigens not represented by Vendor 1, and that the standard panel from Vendor 2 has a 3.1% to 7.2% increased HLA-C representation than Vendor 1. A large contributor to this difference is the absence of HLA-C*07:01 in the standard panel from Vendor 1, since HLA-C*07:01 is found in 13.7% to 24.1% of the population depending on ethnicity. The most similar allele to HLA-C*07:01 that is represented in SAB assays is HLA-C*07:02, where C*07:01 differs by two amino acid substitutions: K66N and S99Y. While position 66 is exposed to the surface and may generate DSA that would not be otherwise detected by the standard panel from Vendor 1, the 66N sequence is a shared epitope also found in HLA-C*15:02, which is included in the panel. However, a single amino acid is not sufficient to define an HLA epitope, so the presence of 66N does not guarantee that a DSA against 66N in the context of HLA-C*07:01 would be detected. Furthermore, if antibodies against HLA-C*15:02 were detected, it is unlikely that these would be assigned as DSA because they would not bind to the HLA-C*07 protein that is in the SAB assays.

For solid organ transplantation, the true prevalence and importance of AMR caused primarily by HLA DSA that were not detected by SAB is unknown. If HLA DSA are present but not detectable, graft rejection or damage may be misattributed to other factors. In this situation, a diagnosis of AMR in the absence of no detected HLA DSA might be incorrectly attributed to non-HLA antibodies, which complicates clinical assessment and treatment. AMR can also occur in the presence of multiple DSA against different epitopes. In such a case, one low titer antibody may be detected while a high titer antibody may be very important but not detectable. Although DSA would be detected, the failure to appreciate the full picture could adversely influence diagnosis, prognosis, and management. It is impractical to develop SAB assays that represent every potential HLA epitope, but it is reasonable to consider the possibility of undetectable DSA against HLA. For example, HLA DSA should not be ruled out for a patient with humoral rejection if the donor has HLA epitopes that are not represented in the SAB assays.

One objective of this study was to determine how these observations are relevant in routine practice by studying our local organ donor population. In our cohort of 60 donors, 26.7% of donors had unrepresented HLA-A, -B, -C, and -DRB1 proteins. All of these have the potential to influence assessment of DSA because antibody binding is influenced by the entire HLA molecule. The same amino acid difference present in different HLA proteins can alter antibody binding characteristics. While unrepresented proteins may contain epitopes that are not found in SAB assays, their amino acid locations suggest that there is variability in both their immunogenicity and the ability of SAB assays to detect DSA against these epitopes. A subset of these donors had epitopes that could generate HLA DSA that may not be detectable by SAB assays (e.g., charge difference, exposed to the surface, and not found in any reagent HLA proteins). Previous studies have suggested that HLA mismatches at the allele-level that are not found with routine low-resolution typing may present as clinically significant DSA after transplantation (13). Our investigation demonstrates that the current dogma suggesting that all clinically significant DSA are detectable by SAB assays should be reconsidered.

A major limitation of this study is that it is limited to HLA-A, -B, -C, and -DRB1 because of the complexity in studying HLA-DQ and HLA-DP proteins which are formed by heterodimers with up to four alpha and beta subunit combinations in a single donor. Although HLA-DR is a heterodimer, the alpha chain is not polymorphic (14). The vast diversity of possible Class II DQ and DP proteins may contribute to diversity in DSA production, further increasing the probability that clinically relevant DSA might not be detected by SAB assays (15, 16).

One of the limitations of this study is that it is based upon HLA allele frequencies determined from self-reported ethnicity. The CIWD catalog consists of aggregated data across multiple donor registries with unstandardized forms of self-reported ethnicity, which introduces inherent uncertainty when this data is used for analysis. Because the ability of SAB assays to detect DSA varies with ethnicity, an accurate understanding of a patient population’s ethnic distribution is helpful in determining when DSA may not be detected. This study was conducted on donor-recipient pairs at Children’s Hospital Los Angeles, where Hispanic and Caucasian ethnicities are the predominant recipients and donors. Consequently, one limitation of the deceased donor analysis is that African American and Asian donors are underrepresented in this study, limiting generalizability to patient populations with different ethnic distributions, but at the same time demonstrating the importance of recognizing these factors in a center’s particular population. Additionally, data on ethnicities of deceased donors were self-reported, which may not always represent the genetic ancestry of each donor (17). Another limitation is that it is unclear how well the sample population in the CIWD database agrees with Hardy-Weinberg equilibrium. Smaller-scale regional studies have shown variable deviations in Hardy-Weinberg equilibrium (18–20), and the current study uses combined data from worldwide donor registries.

A frequent misconception is that DSA can be excluded if a deceased donor meets the Organ Procurement and Transplantation Network (OPTN) criteria for a 0-antigen mismatch. Our case study illustrates this risk, in which a donor-recipient pair classified as 0-ABDR mismatch had multiple allele-level mismatches, one of which involved an HLA protein not found in standard panels from either vendor. The case reported here shows that patients receiving organs from 0-antigen mismatched donors could develop DSA that might not be detected using SAB assays. The fact the HLA-A, B, and -DR are supposedly well-matched increases the potential for a humoral response against HLA epitopes that may not be represented in the SAB assays (13). Additionally, the patient can develop DSA against proteins from the other HLA loci (e.g., HLA-C, DQ, and DP).

When DSA are not detected despite suspected AMR, high resolution HLA typing is essential to determine if donor antigens are not present in SAB assays. Studies that rely upon inferred HLA types may be inaccurate because this approach is biased toward assigning high frequency alleles (21). For instance, Lehmann et al. found that 64.1% of their patient sample had HLA mismatches that were identified with allele-level HLA typing but would otherwise have been missed by low-resolution typing (22). This example illustrates that the risk of undetected DSA may be underestimated in routine clinical practice. The possibility of undetected DSA should be considered in current transplant protocols where HLA mismatches are determined using low resolution HLA types (21).

Our study has important conceptual implications for virtual crossmatching and transplant treatment guidelines. These implications are that 1) SAB assays may not definitively rule out the presence of DSA, 2) unrepresented HLA proteins are fairly frequent, and 3) allele-level HLA typing may be needed to fully understand the likelihood of unrepresented antigens in the panel used in a given laboratory. The possibility of undetected DSA should be considered in suspected AMR without detection of HLA antibodies, especially in ethnically diverse patient populations. Cell-based crossmatches using donor cells could compensate for this limitation, but cannot be used unless viable donor cells are available.

In conclusion, SAB assays offer many advantages over previous technologies for detecting HLA antibodies, but a risk of failure to detect DSA using SAB assays still exists. The risks of undetectable DSA are increased with non-European donor populations and vary between reagent panels. Allele-level HLA typing can increase the accuracy of HLA histocompatibility determination and is useful for assessing the risk of undetectable DSA.

## Data availability statement

The original contributions presented in the study are publicly available. This data can be found here: https://figshare.com/articles/dataset/_b_HLA_Typing_for_Deceased_Donors_b_/23820606/1.

## Ethics statement

This study was deemed exempt by the Children’s Hospital Los Angeles Human Subjects Protection Program and Institutional Review Board (CHLA-23-00143). This study does not involve human subjects per the US Department of Health and Human Services regulation 45 CFR 46.102 (e) (1). This study does not use, study, generate, or analyze identifiable private information or biospecimens.

## Author contributions

JQ: Data curation, Investigation, Visualization, Writing – original draft, Writing – review & editing, Formal Analysis. KK: Data curation, Formal Analysis, Investigation, Writing – review & editing, Visualization. NF: Data curation, Formal Analysis, Investigation, Visualization, Writing – review & editing. JM: Conceptualization, Data curation, Formal Analysis, Funding acquisition, Investigation, Methodology, Supervision, Writing – original draft, Writing – review & editing. RL: Data curation, Formal Analysis, Investigation, Writing – review & editing, Conceptualization, Funding acquisition, Methodology, Supervision, Writing – original draft. MW: Data curation, Formal Analysis, Investigation, Writing – review & editing. LB-L: Conceptualization, Data curation, Formal Analysis, Funding acquisition, Investigation, Methodology, Project administration, Supervision, Visualization, Writing – original draft, Writing – review & editing.
