# Supplementary material for: HLA diversity in ethnic populations can affect detection of donor-specific antibodies by single antigen beads
Source: Front Immunol. 2023 Nov 23;14:1287028. doi: 10.3389/fimmu.2023.1287028 (PMC10701672; doi:10.3389/fimmu.2023.1287028)
Supplement: Supplementary file 1 [file DataSheet_1.pdf]

Supplementary Material

HLA Diversity in Ethnic Populations Can Affect Detection of Donor-Specific Antibodies by Single Antigen Beads

Justin C. Quon, Kelli Kaneta, Nicholas Fotiadis, Jondavid Menteer, Rachel M. Lestz, Molly Weisert, Lee Ann Baxter-Lowe*

*** Correspondence:** Lee Ann Baxter-Lowe: lbaxterlowe@chla.usc.edu

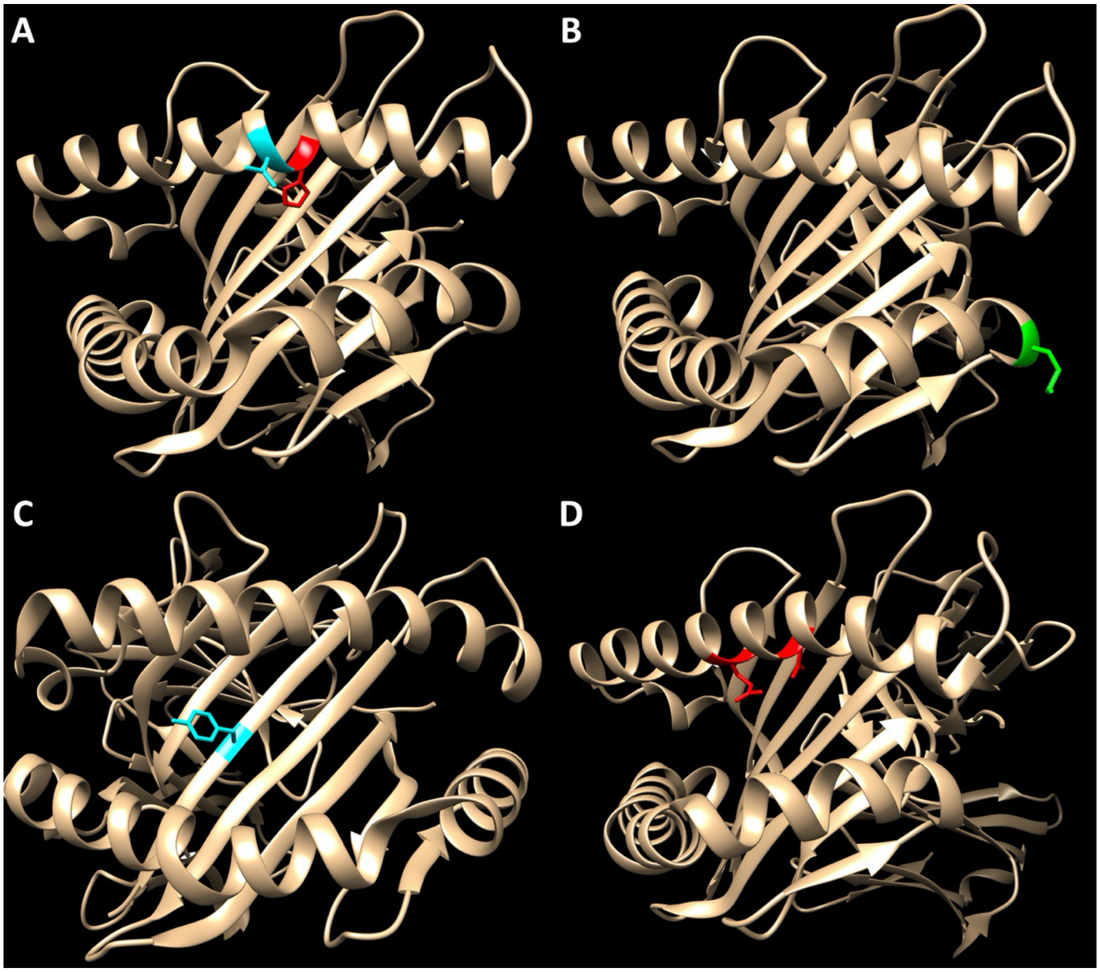

Supplementary Figure 1. Green residues indicate no charge difference between the unrepresented protein and the most similar reagent protein. Blue residues indicate a polarity difference but no charge difference. Red residues indicate an overt charge difference at that position. **(A)** Structure of HLA-A*02:01 with residues 73T (blue) and 74H (red) corresponding to unrepresented HLA-A*02:11. **(B)** Structure of HLA-A*02:01 with residue 138M (green) corresponding to unrepresented HLA-A*02:164. **(C)** Structure of HLA-A*24:02 with residue 7Y corresponding to unrepresented HLA-A*24:25. **(D)** Structure of HLA-A*68:01 with residues 70Q (red) and 74D (red) corresponding to unrepresented HLA-A*68:05.

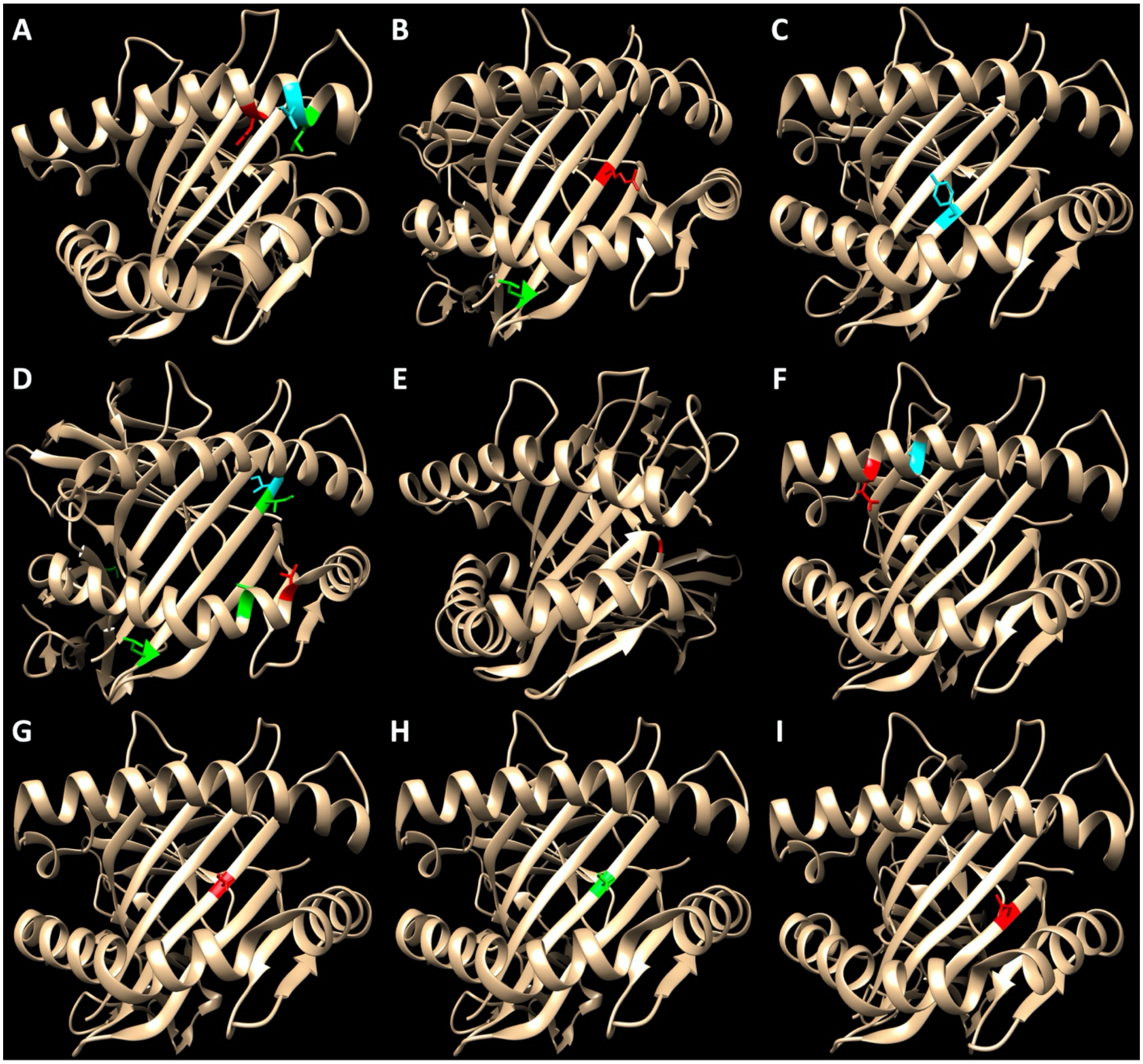

Supplementary Figure 2. Green residues indicate no charge difference between the unrepresented protein and the most similar reagent protein. Blue residues indicate a polarity difference but no charge difference. Red residues indicate an overt charge difference at that position. **(A)** Structure of HLA-B*27:05 with residues 77D (red), 80I (blue), and 81L (green) corresponding to unrepresented HLA-B*27:02. **(B)** Structure of HLA-B*35:01 with residues 97R (red) and 103L (green) corresponding to unrepresented HLA-B*35:17. **(C)** Structure of HLA-B*35:01 with residue 99Y (blue) corresponding to unrepresented HLA-B*35:23. **(D)** Structure of HLA-B*35:01 with residues 94I (blue), 95I (green), 103L (green), 152V (red), and 194V (green) corresponding to unrepresented HLA-B*35:43. **(E)** Structure of HLA-B*35:01 with residue 120G (red) corresponding to the unrepresented HLA-B*35:116. **(F)** Structure of HLA-B*40:02 with residues 63E (red) and 67S (blue) corresponding to unrepresented HLA-B*40:08. **(G)** Structure of HLA-B*40:02 with residue 97S (red) corresponding to unrepresented HLA-B*40:11. **(H)** Structure of HLA-B*40:02 with residue 97S (green) corresponding to unrepresented HLA-B*40:225. **(I)** Structure of HLA-B*44:02 with residue 116D corresponding to unrepresented HLA-B*44:05.

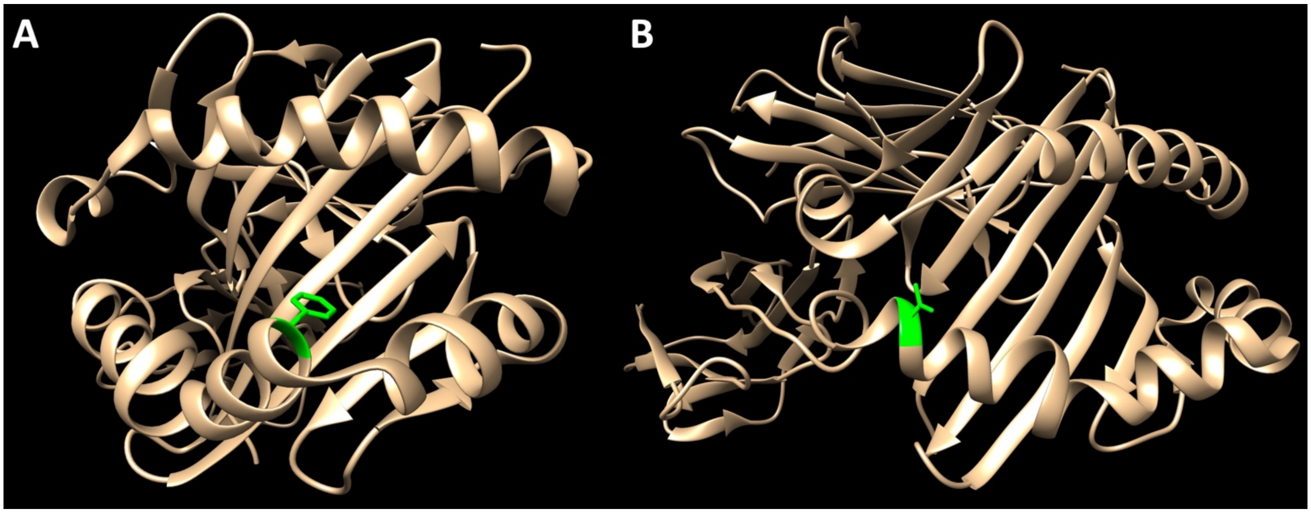

Supplementary Figure 3. Green residues indicate no charge difference between the unrepresented protein and the most similar reagent protein. **(A)** Structure of DR11 with residue 67F (green) corresponding to unrepresented HLA-DRB1*11:02. **(B)** Structure of DR11 with residue 85V (green) corresponding to unrepresented HLA-DRB1*11:06.
